# Demonstration of asymmetric electron conduction in pseudosymmetrical photosynthetic reaction centre proteins in an electrical circuit

**DOI:** 10.1038/ncomms7530

**Published:** 2015-03-09

**Authors:** Muhammad Kamran, Vincent M. Friebe, Juan D. Delgado, Thijs J. Aartsma, Raoul N. Frese, Michael R. Jones

**Affiliations:** 1 Leiden Institute of Physics, Leiden University, Niels Bohrweg 2, 2333 CA Leiden, The Netherlands; 2 Department of Physics and Astronomy, LaserLaB Amsterdam, VU University Amsterdam, De Boelelaan 1081, 1081 HV Amsterdam, The Netherlands; 3 School of Biochemistry, University of Bristol, Medical Sciences Building, University Walk, Bristol BS8 1TD, UK

## Abstract

Photosynthetic reaction centres show promise for biomolecular electronics as nanoscale solar-powered batteries and molecular diodes that are amenable to atomic-level re-engineering. In this work the mechanism of electron conduction across the highly tractable *Rhodobacter sphaeroides* reaction centre is characterized by conductive atomic force microscopy. We find, using engineered proteins of known structure, that only one of the two cofactor wires connecting the positive and negative termini of this reaction centre is capable of conducting unidirectional current under a suitably oriented bias, irrespective of the magnitude of the bias or the applied force at the tunnelling junction. This behaviour, strong functional asymmetry in a largely symmetrical protein–cofactor matrix, recapitulates the strong functional asymmetry characteristic of natural photochemical charge separation, but it is surprising given that the stimulus for electron flow is simply an externally applied bias. Reasons for the electrical resistance displayed by the so-called *B*-wire of cofactors are explored.

## Abstract (web summary)

Photosynthetic reaction centres have been proposed for applications in bioelectronics. Here, the authors examine electron transport through the reaction centre from *R. sphaeroides* using conductive AFM, observing asymmetric conductance along only one cofactor wire under an applied bias.

## Graphical abstract

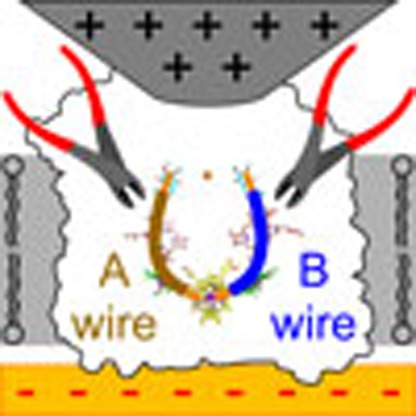

In the quest to fabricate nanoscale electronic circuits, electrically conductive peptides and redox proteins show potential as single-molecule components such as switches and gates^1^,^2^,^3^,^4^,^5^. Among the many attractions of proteins for nanoscale electronics are their enormous natural variety and the scope to use the tools of protein and genetic engineering to fabricate new biocompatible components with defined electrical and structural properties. The need to understand how electrons tunnel through individual protein molecules under an applied bias has been a driver in the development of techniques such as conducting atomic force microscopy (C-AFM) and scanning tunnelling microscopy that can probe the electrical properties of single molecules fixed between a substrate and an electrically conductive probe tip^6^. These techniques are providing insights into how the electrical properties of a protein are defined by its composition and structure^1^,^2^,^3^,^4^,^5^,^7^.

Among the diversity of naturally conductive proteins, photosynthetic reaction centres (RCs) offer a unique combination of properties that could lend themselves to multiple applications in molecular-scale electronics. Under illumination, RCs act as solar batteries, using harvested light energy to separate charge between an electron donor at one end of the protein and an acceptor at the other^8^,^9^,^10^,^11^. This multi-step process occurs with a very high quantum efficiency (charges separated across the RC protein per photon absorbed) and, in biology, creates a potential difference that powers an external linear or cyclic electron transfer system. This fundamentally important energy conversion process has been extensively characterized, in particular in the RC from the purple bacterium *R*. (*Rba*.) *sphaeroides*^8^,^9^,^10^, and it is well established that RCs of various kinds can be interfaced with electrode materials for the generation of photocurrents in biohybrid devices^12^,^13^,^14^. The particular *Rba. sphaeroides* RC used in the present work has been efficiently interfaced with unfunctionalized gold electrodes^15^, used to generate direct or alternating photocurrents^15^,^16^ and in addition to photovoltaics *per se* it has been used for applications such as biosensing^17^.

The structural and energetic attributes that underpin the high quantum efficiency of photochemical charge separation by RCs also result in diode-like behaviour. In all RCs, photoexcitation of a (bacterio)chlorophyll species at one side of the photosynthetic membrane results in the separation of charge along a ‘wire’ of redox cofactors. Transfer of the electron along this wire across the membrane is associated with a drop in reduction potential of at least 1 V, such that forward electron transfer is very strongly favoured. Application of C-AFM to oriented *Rba. sphaeroides* RCs in the dark and under an applied external bias of appropriate polarity has revealed that current will flow through the protein from the electron donor side to the electron acceptor side, with the current amplitude growing with increasing bias, but unlike a great many peptides or redox proteins little or no current is seen if a reverse bias is applied, irrespective of its magnitude^18^,^19^,^20^. The same phenomenon has been observed to varying degrees when the RC is associated with an LH1 antenna protein^21^,^22^ and in Photosystem I from oxygenic photosynthetic organisms^23^,^24^.

An open question is the route or routes taken by electrons that tunnel through the *Rba. sphaeroides* RC under a favourable external bias. Following the structural blueprint on which most photosynthetic RCs are based^25^,^26^,^27^,^28^, in the *Rba. sphaeroides* complex a protein scaffold displaying twofold pseudo-symmetry holds in place two symmetrical wires of redox cofactors that span the membrane^29^,^30^,^31^. It is well established that only one of these wires, usually referred to as ‘active’ or ‘A’, conducts photochemical charge separation through the protein^32^. This is initiated by formation of the first singlet electronic state of a ‘special pair’ (*P*) of bacteriochlorophylls (BChls) located close to one side of the membrane (*P*-side). A dissociable ubiquinone (*Q*_B_) on the opposite side (*Q*-side) is then reduced in a four-step process involving a ‘redox wire’ comprising a monomeric BChl (*B*_A_), a bacteriopheophytin (BPhe—*H*_A_) and a non-dissociable ubiquinone (*Q*_A_)^8^,^9^,^10^,^33^,^34^,^35^. No significant level of electron transfer is observed along the symmetrical *B*-wire of cofactors connecting *P* directly to *Q*_B_^32^,^36^. This strong functional asymmetry stems from differences in the reduction potential of equivalent cofactors in the two wires, differences in electronic coupling between the special pair excited electronic state (P*) that triggers electron transfer and the two potential acceptors *B*_A_ and *B*_B_, and possibly also differences in reorganization energy along the two wires^32^,^36^,^37^,^38^. The structural reasons for these asymmetries are not fully understood, but they stem from multiple differences in detailed structure of the two polypeptides, PufM and PufL, which form the scaffold that holds the two cofactor wires in place^30^,^31^. These polypeptides have a similar fold but are only ~33% identical^39^, facilitating microscopic asymmetry in a structure with macroscopic symmetry.

The work described below addresses the mechanism of electron conduction through the *Rba. sphaeroides* RC under an applied external bias. Specifically, we examine whether electron flow through the protein follows the striking asymmetry displayed by photochemical charge separation, or can proceed via either of the cofactor wires that span the protein. The same question hangs over photocurrent generation by RCs immobilized on electrodes—there is an assumption that this is a consequence of charge separation along the *A*-wire only, but as yet no proof of this has been presented. These questions were addressed by selectively breaking each of the two cofactor wires in the *Rba. sphaeroides* RC by replacing an alanine side chain by a bulkier tryptophan at locations that cause the RC to assemble either without the quinone (*Q*_A_) that forms one end of the *A*-wire^40^ or the BPhe (*H*_B_) that is located halfway along the pseudosymmetrical *B*-wire^41^. The ability of these mutated RCs to conduct current under an applied potential was examined by C-AFM and their ability to generate photocurrents was examined by photochronoamperometry. The findings throw new light on the mechanisms by which electrons are conducted through RCs under illumination or an externally applied potential in biohybrid architectures, revealing that under both conditions the conduction of electrons is highly asymmetric.

## Results

### Engineered RCs with broken electron conduction wires

**Figure 1:**
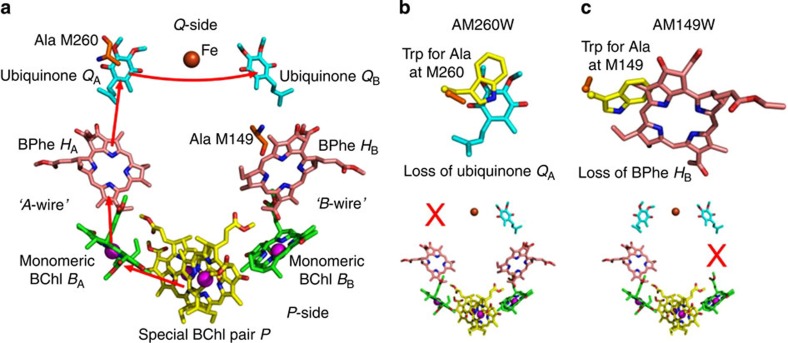
Structures of wild-type and engineered RCs. (**a**) Two wires of cofactors connect the *P*-side (positive terminus) and *Q*-side (negative terminus) of the wild-type RC. Photoexcitation of the *P* dimer triggers electron flow along the *A*-wire (red arrows) forming, sequentially, *P*^+^*B*_A_^−^, *P*^+^*H*_A_^−^ and *P*^+^*Q*_A_^−^ radical pairs. Electrons exit the complex via the dissociable *Q*_B_ ubiquinone. (**b**) Overlay of the X-ray crystal structures of the wild-type and AM260W mutant RCs shows that replacement of Ala M260 by Trp prevents incorporation of the *Q*_A_ ubiquinone due to steric overlap (top), breaking the *A*-wire (bottom). (**c**) Similarly, replacement of Ala M149 by Trp prevents incorporation of the *H*_B_ BPhe (top), breaking the *B*-wire (bottom). Key for all panels: nitrogen—blue, oxygen—red, cofactor carbons—yellow, green, pink or cyan, protein carbons—orange (wild-type RC) or yellow (mutant RC), magnesium—magenta spheres, iron—brown sphere.

RC-enriched membrane fragments were prepared from strains of *Rba. sphaeroides*, which lacked light-harvesting pigment proteins (see Methods). In addition to a strain expressing wild-type RCs, two variants were used in which either an alanine at the 260 position in the PufM polypeptide was changed to tryptophan (AM260W) or an alanine at the 149 position was changed to tryptophan (AM149W). The arrangement of electron transfer cofactors in the wild-type *Rba. sphaeroides* RC in two pseudo-symmetrical membrane-spanning wires is shown in Fig. 1a. For both engineered RCs, a combination of X-ray crystallography and spectroscopy has shown that the much bulkier tryptophan residue takes up volume normally occupied by an adjacent cofactor (Fig. 1b,c), preventing incorporation of that cofactor into its binding pocket during assembly of the RC^40^,^41^,^42^,^43^. Hence, it is clearly established that the *Q*_A_ ubiquinone is not present in the AM260W RC^40^,^42^, breaking the *A*-wire of cofactors (Fig. 1b), and the *H*_B_ BPhe is not present in the AM149W RC^41^,^43^, breaking the *B*-wire of cofactors (Fig. 1c).

### Photocurrent generation by RCs

**Figure 2:**
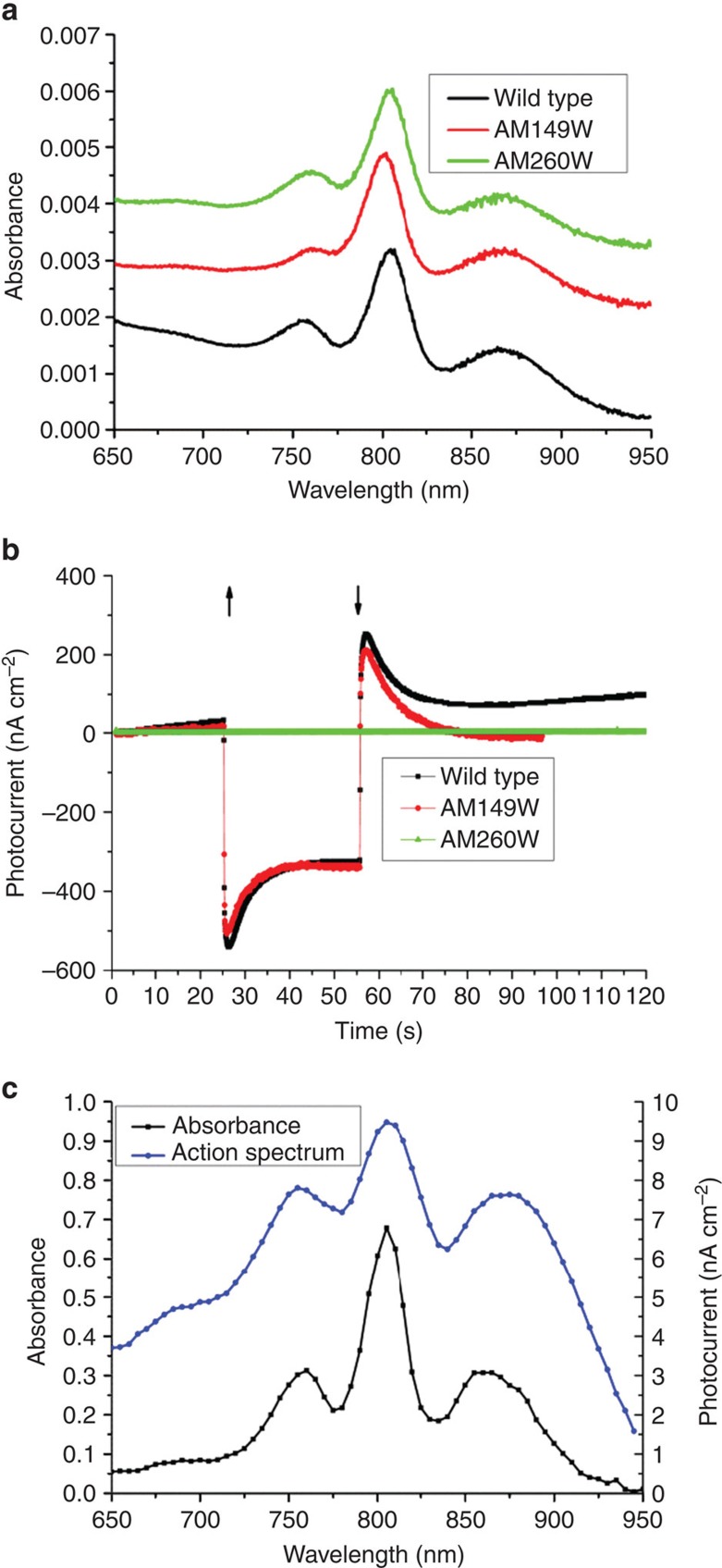
Photochronoamperometry of RC-rich membranes. (**a**) Absorbance spectra of RC-rich membranes adhered to gold working electrodes. (**b**) Photocurrent response of coated electrodes during 30 s of illumination (↑—light on,↓—light off). (**c**) Comparison of absorbance spectrum at 5 nm intervals with the action spectrum of photocurrent density for adhered membranes with wild-type RCs.

For photochronoamperomety, RC-rich membrane fragments were adsorbed onto an unfunctionalized gold electrode comprising a 25-mm diameter glass coverslip coated with a flat (2–3 Å root-mean-square roughness) layer of gold^44^. The absorbance spectrum of adsorbed wild-type RCs (Fig. 2a) indicated a coverage of around 2.7 × 10^12^ RCs per cm^2^, which corresponded to 60%–70% of a full monolayer given the known cross-sectional area of a single RC. Coated electrodes were immersed into a photoelectrochemical cell containing buffer and fitted with a platinum wire counter electrode and saturated calomel reference electrode. Maximal cathodic photocurrents were obtained in the presence of 800 μM ubiquinone-0 and 80 μM cytochrome *c* as mediators (Fig. 2b). Under illumination from a light emitting diode (LED) centred at 850 nm (intensity 23 mW cm^−2^ at the electrode surface), a steady photocurrent in the region of 350 nA cm^−2^ was obtained from adsorbed wild-type RCs after an initial transient of higher current that decayed over ~15 s (Fig. 2b, black). The transient reverse current observed after turning off the excitation light is probably due to dissipation of stored charges in the system, as discussed previously^16^,^45^. Measurements with monochromatic excitation showed that the current amplitude tracked the absorbance spectrum of the RC in the near infrared (Fig. 2c).

To investigate for the first time which of the two wires of cofactors contributed to photocurrent generation in a device setting, electrodes were also prepared with adsorbed membrane fragments containing either AM260W or AM149W RCs. The coverage of RCs at the electrode surface was similar to that obtained with wild-type RCs (Fig. 2a). The absorbance spectrum of the AM149W RC (Fig. 2a, red) had a relatively low band amplitude at 760 nm, consistent with the absence of one of the two BPhe cofactors^41^. Electrodes coated with AM149W RCs were able to support photocurrents at a level consistent with that seen in the wild-type RC (Fig. 2b, red compared with black), showing that breaking the *B*-wire had no significant impact, but no current was obtained after breaking the *A*-wire in the AM260W RC (Fig. 2c, green). The implication of this is that the source of the observed photocurrent in the wild-type RC is charge separation along the *A*-wire of cofactors to form the radical pair *P*^+^*Q*_A_^−^ (Fig. 1a).

### Conduction by RCs under an applied bias

**Figure 3:**
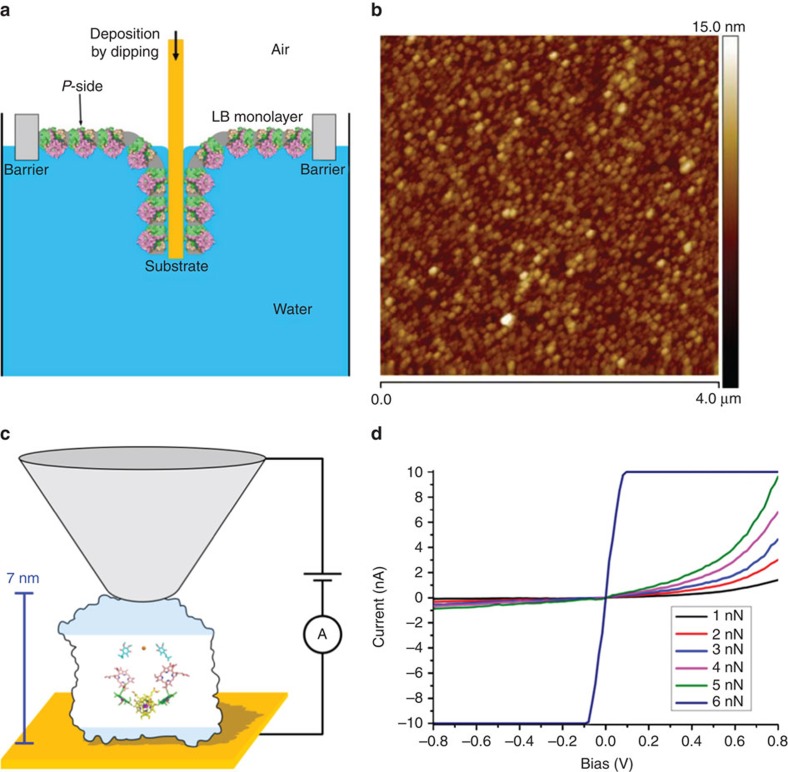
Deposition of LB films and conductive AFM. (**a**) Schematic of the deposition of an LB monolayer of RC-rich membranes by dipping to achieve a uniform *P*-side down orientation. (**b**) Tapping mode AFM of a deposited LB film. (**c**) Schematic of the conductive AFM measurement; a potential ramp is applied between the AFM tip (not to scale) and the gold substrate at a range of applied tip forces, the tunnelling junction being formed by the RC-rich LB film. The proportion of the RC residing outside the membrane is indicated by shading; the two cofactor wires (shown as in Fig. 1a) span the membrane-embedded region. (**d**) Current-–voltage profiles for deposited wild-type RCs at varying tip forces.

The ability of these RCs to conduct current under an applied external bias was examined using C-AFM. A flat and uniformly oriented experimental sample is particularly important for such measurements, as RCs with random orientation do not show diode-like behaviour^19^,^24^. Accordingly the Langmuir–Blodgett (LB) technique was used to deposit RC-rich membrane fragments onto the same type of unfunctionalized gold electrode as was used for photocurrent measurements. A solution of RC-rich membranes (1 mg RC per ml) was spread on the air–liquid interface in an LB trough, the monolayer compressed to a surface pressure of 35 mN m^−1^ and gold electrodes coated by dipping into the sub-phase at constant pressure (Fig. 3a). The topography of the deposited RC-rich membrane fragments was examined using tapping-mode AFM under ambient conditions, revealing closely packed structures of regular size with a reasonably uniform height distribution (Fig. 3b).

The electrical properties of the deposited monolayer were investigated in the tunnelling junction formed by a C-AFM tip and the gold substrate (see Fig. 3c for a schematic). Current–voltage profiles were measured at various locations, the contact force exerted by the probe tip being controlled by AFM feedback in contact mode. A negligible current was obtained at a negative applied bias (Fig. 3d) with a nonlinear rise of the tunnelling current at increasingly positive bias (electrons flowing through the protein from the substrate to the tip). In line with previous observations on electron tunnelling through redox proteins^46^, the tunnelling current at positive bias increased with applied force (Fig. 3d). Above a maximum tip force of 5 nN, the response became ohmic, presumably due to breakdown of the membrane and direct contact between the conducting tip and underlying surface. Notably, the diode junction behaviour was retained up to the 5 nN maximum, with no substantial reverse current at the negative bias. Current–voltage measurements at different locations on the layer of adsorbed membranes produced similar profiles, the only difference being some variation (up to 25%) in the absolute magnitude of the response (data not shown). This consistency indicated that the RCs in the layer of membrane fragments had a uniform orientation, the polarity of the bias required for a tunnelling current, suggesting that this orientation placed the RC *P*-side (Fig. 1a) closest to the gold substrate (as depicted in Fig. 3a,c).

**Figure 4:**
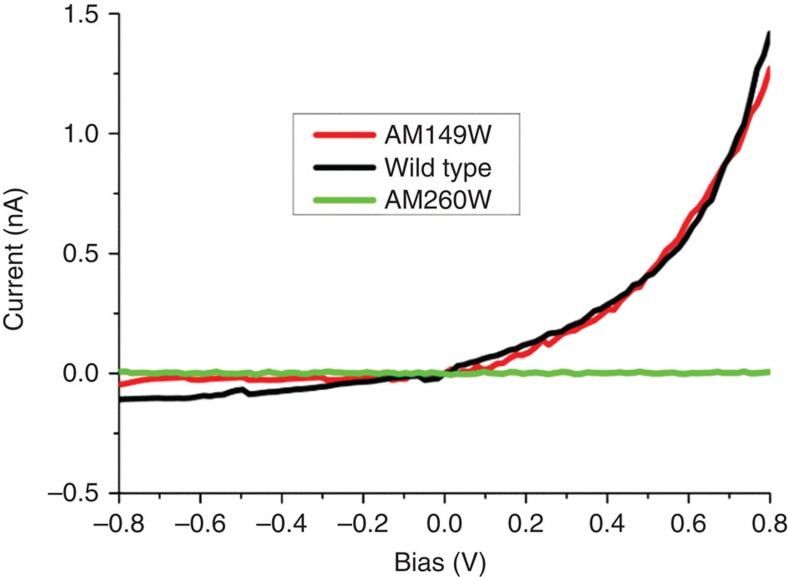
Tunnelling of electrons across engineered RCs. Current voltage profiles for wild-type and engineered RCs at an applied tip force of 1 nN.

Current–voltage profiles were also measured for LB-deposited membranes containing either AM260W or AM149W RCs (Fig. 4). Remarkably, no tunnelling current was obtained with AM260W RCs at any applied force up to 5 nN, at which point the ohmic response was obtained. This demonstrated that the intact *B*-wire of cofactors was not capable of conducting measureable amounts of current at the minimal tip force and could not be induced to conduct current by increasing that force. This lack of participation by the *B*-wire was reinforced in measurements with the AM149W RC, where the current obtained was similar in magnitude to that obtained with wild-type RCs, as was its response to increased positive bias (Fig. 4, red compared with black) and increased tip force up to 5 nN (not shown).

## Discussion

Key to the future manipulation of proteins as electrical components is an understanding of their conductive and resistive properties in a biohybrid setting. It is well established that purple bacterial RCs can be interfaced with conducting electrodes for the generation of photocurrents and much is known about suitable combinations of electrode materials, linkers and redox mediators, but relatively little is known about the actual mechanism through which photocurrents are generated. Most of what has been concluded in recent years has been based on indirect evidence such as the relative redox or vacuum potentials of different components in a particular electrochemical cell, the direction of current flow under different potentials applied to the working electrode, the requirement for specific types of redox mediator and the action of a limited number of available inhibitors^12^,^13^,^14^,^15^,^16^,^17^,^47^,^48^,^49^,^50^,^51^,^52^,^53^.

In the present report, protein engineering has been used to establish that photocurrent generation by *Rba. sphaeroides* RCs interfaced to gold electrodes is dependent on the conventional mechanism of charge separation in which an electron traverses the membrane exclusively via the *A*-wire to form the radical pair *P*^+^*Q*_A_^−^. The lack of a photocurrent in the *Q*_A_-deficient AM260W RC, together with the lack of a measureable impact of the AM149W mutation, argues against any contribution of photochemical charge separation along the *B*-wire to the observed photocurrent, even when the *A*-wire is broken.

**Figure 5:**
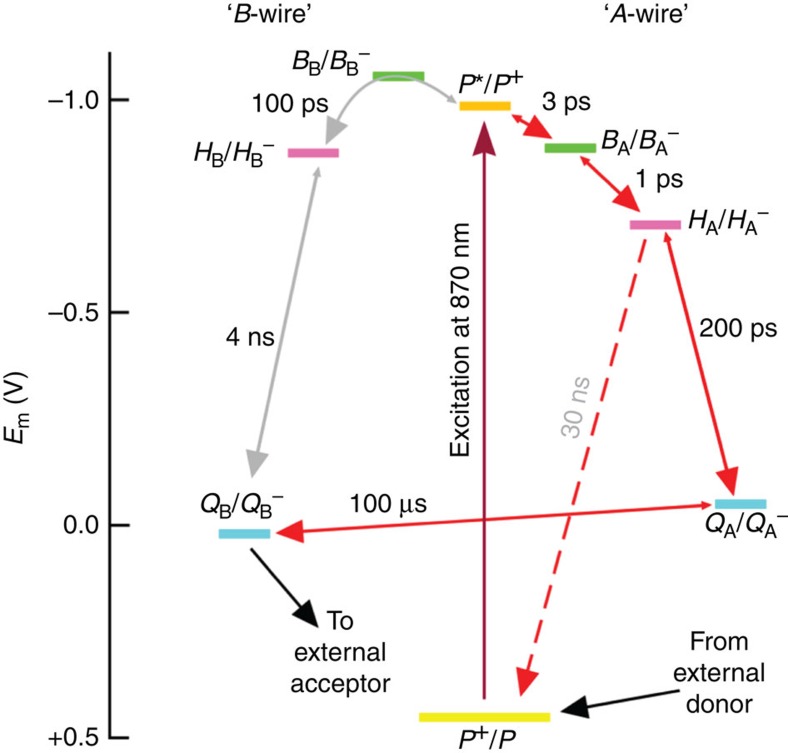
Redox basis for asymmetric photochemical charge separation. Plot shows the mid-point potentials of redox couples involved in *A*-wire photochemical charge separation (right) and equivalent states involving *B*-wire cofactors (left).

The basis of asymmetric charge separation and the blockage in photocurrent generation by the AM260W mutation is explored in Fig. 5, which considers relevant redox potentials on the two cofactor wires. Asymmetric charge separation following photo-excitation of the *P* special pair (purple arrow) is accounted for, in part (see below), by *B*_B_ having a more negative reduction potential than its counterpart *B*_A_ or the electron donor P*, such that fast stepwise electron transfer via *B*_A_^−^ and *H*_A_^−^ to *Q*_A_ (red arrows) outcompetes slow 100 ps electron transfer to *H*_B_ (probably by superexchange involving *B*_B_—grey bent arrow)^36^,^37^. Tuning of redox potentials, and hence free energies of radical pairs formed along the two wires, has a variety of origins including specific hydrogen bond or charge–dipole interactions^36^,^37^, differences in the conformations of BChl substituent groups^54^, differences in effective dielectric constant along the two cofactor wires^55^ and asymmetries in static electric fields arising from charged amino acids^56^. In the AM260W mutant, the absence of *Q*_A_ means that electrons that travel to *H*_A_ recombine with *P*^+^ (as the main process among other, slower loss routes), as shown by the red dashed arrow in Fig. 5. However, the initial branching is not affected by the absence of *Q*_A_, and so enhanced electron flow along the *B*-wire is not seen. Light therefore mainly powers a futile cycle of formation and recombination of the *P*^+^*H*_A_^−^ radical pair on the *A*-wire and no photocurrent is produced by the AM260W RC. It should be noted that other factors also contribute to the differences in rate of equivalent electron transfer steps in the two wires, including asymmetries in electronic coupling between neighbouring cofactors^38^.

Although the finding that *Rba. sphaeroides* RCs perform highly asymmetric photochemical charge separation at an electrode surface is not necessarily surprising, more of a surprise was the finding from C-AFM that conduction under an externally imposed potential difference also showed this behaviour. This electron conduction operates by a different mechanism that uses cofactor electronic levels that lie between the Fermi energies of the conducting tip and substrate^1^,^2^,^3^,^4^,^5^, presumably either the lowest unoccupied molecular orbitals of the cofactors that form the *A*-wire, or the highest occupied molecular orbitals of these cofactors in a mechanism involving hole transfer initiated at the *Q*-side of the RC.

For wild-type RCs deposited on the substrate from an LB film, electron tunnelling from the substrate to the tip was consistently seen at positive bias potentials, which indicates that the RCs were uniformly oriented with the *P*-side closest to the gold substrate. Although LB films of purified *Rba. sphaeroides* RCs and RC/antenna complexes have been studied by many laboratories^13^,^45^,^57^,^58^,^59^, and films of intact native membranes constructed^57^, to our knowledge LB films of membranes enriched in RCs have not been formed previously. This was possible in the present study through the use of engineered bacterial strains that lack the light-harvesting complexes that dominate the photosynthetic membrane of the wild-type bacterium^60^. The RC-rich membrane fragments isolated from these strains formed LB films that, when imaged by AFM, comprised regularly sized membrane patches of ~50 nm diameter. Based on the uniformity of the response in C-AFM measurements, we conclude that these membrane patches had a common orientation in the LB film with the *P*-side of the RC adjacent to the substrate (Fig. 3a). As the substrate was coated by dipping this orientation implies that the *Q*-side of the RC was exposed to the aqueous phase in the LB film, which is consistent with its larger surface area exposed on this side of the membrane due to the protruding H-polypeptide (Fig. 3a, and blue shaded regions in Fig. 3c). Given the dimensions of a single RC, roughly a 7 × 5 nm ellipse in the plane of the membrane, it is possible that each membrane patch could accommodate several RCs. In addition, given the 15-nm radius of curvature of the C-AFM tip, it is likely to be that the measured tunnelling current was supported by several uniformly oriented RCs. A current of 1.4 nA was measured at an applied bias of 0.8 V, and if it is assumed that this current was mediated by five RCs then this would correspond to conduction of one electron per 570 ps. This is compatible with the well-established lifetimes for membrane-spanning charge separation in this RC^8^,^9^,^10^.

In C-AFM of a protein, the size of the current is expected to depend on the bias voltage (expressed tip relative to substrate), the length of the protein along the axis between tip and substrate (Fig. 3c), the height of the tunnelling barrier and the contact area between the probe and the protein(s) that are providing the electrical connection to the substrate^1^,^2^,^3^,^4^,^5^. The barrier height is dependent on the composition and structure of the protein, and will be affected by details such as the spacing and electrochemical properties of bound cofactors. As outlined in the Introduction, the diode-like behaviour of the RC can be accounted for by the large potential drop involved in transferring an electron through the central region of the protein from the *P*-side to the *Q*-side (Fig. 5). The data outlined above show that a positive bias induces electron flow along the *A*-wire, and in an RC oriented with the *P*-side closest to the substrate this would be occurring in an energetically favourable direction. In contrast, a negative bias would need to overcome the difference of ~1 V between the reduction potentials of the quinones and those of the cofactors near the *P*-side of the RC (Fig. 5), and so is not observed even when the tip force is increased to the maximum attainable value before the integrity of the system breaks down. Without the participation of the cofactors, there should not be significant electron tunnelling across a 7-nm thickness of protein.

More difficult to understand is the lack of any evidence for electron conduction along the *B*-wire under an applied bias. A trivial explanation could be a lack of the *Q*_B_ ubiquinone in AM260W RCs, but this can be discounted. In describing its X-ray crystal structure, McAuley *et al*.^42^ commented on the unusually high degree of occupancy of the *Q*_B_ site in the purified AM260W RC and the region around the *Q*_B_ pocket was unaffected by the changes in structure induced by the alanine to tryptophan mutation near the *Q*_A_ site^42^. In addition, the experimental material used in the present work comprised patches of photosynthetic membrane rather than purified protein; photosynthetic membranes are known to contain high concentrations of ubiquinone, to mediate intercomplex electron transfer at high rates, and so the AM260W RC resides in a quinone-rich local environment.

A possible explanation for the inactivity of the *B*-wire is that conduction is initiated by population of the lowest unoccupied molecular orbital of the special pair of BChls to form a *P* anion (*P*^−^), and that subsequent transfer to either *B*_A_ or *B*_B_ is subject to some or all of the same factors that dictate asymmetry during light-activated charge separation, with formation of *B*_A_^−^ being very strongly favoured due to asymmetries in electronic coupling, local dielectric constant and/or electron transfer rates. *P*^−^ is a radical that is not relevant to photosynthesis and, to our knowledge, the *P*^−^/*P* reduction potential has not been measured or estimated through calculation. However, the experimental observation that both *B*_A_ and *B*_B_ can be fully reduced without any significant reduction of *P* indicates that its reduction potential must be significantly more negative than that of either *B*_A_ or *B*_B_^61^,^62^. In the absence of *Q*_A_, electrons that are conducted along the shortened *A*-wire cannot exit the protein via the *Q*_B_ site as the distance is too large between *H*_A_ and *Q*_B_ to support an appreciable rate of electron transfer between the two (Fig. 1a). As a result, electrons would remain trapped on the *A*-wire centres. An interesting question that follows from this is why, at a point where *H*_A_ and possibly *B*_A_ and *P* are reduced, would electrons not travel along the less-favourable, but intact, *B*-wire to produce a measureable tunnelling current. A possible answer is that the presence of negative charges along the partial *A*-wire would lower the reduction potential of *P*/*P*^−^ to a point where the reduction of *P* by the substrate becomes unfavourable. A related possibility would be a lowering of the potential of *B*_B_/*B*_B_^−^ relative to *P*/*P*^−^ such that the first step of conduction along the *B*-wire becomes unfavourable.

**Figure 6:**
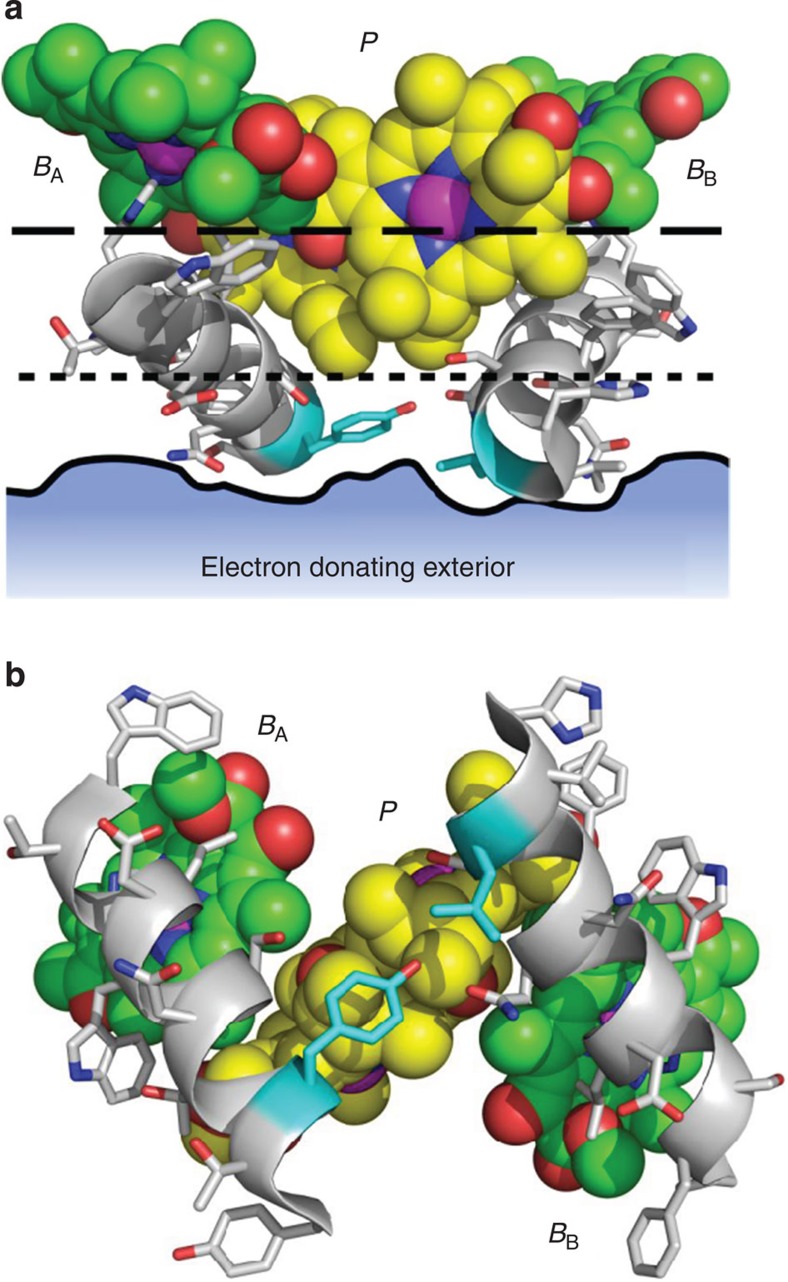
Electron ingress at the *P*-side of the RC. (**a**) The macrocyles of the *P*, *B*_A_ and *B*_B_ BChls are shown as spheres with atom colours as for Fig. 1a. The edges of the macrocycles of the *P* BChls (marked by the dotted line) are located closer to the surface of the protein (solid line) than those of the *B*_A_ and *B*_B_ BChls (dashed line). The solid line shows the boundary of the protein surface in a vertical plane corresponding to the amino acids highlighted with cyan carbons. (**b**) View from below of the structural elements shown in **a**. The *P* BChls are separated from the adjacent aqueous phase by only a single layer of amino acids (highlighted with cyan carbons), whereas the *B*_A_ and *B*_B_ Bchls are more deeply buried within the protein, each overlaid by an amphipathic α-helix. Key: nitrogen—blue, oxygen—red, protein carbons—white.

An underlying implication of the above arguments is that electron injection into the RC by the conductive substrate occurs only via *P*^−^ and not by direct transfer to *B*_A_ or *B*_B_, and, as a result, subsequent electron conduction is influenced by the asymmetry in the electrical connections between *B*_A_/*P* and *B*_B_/*P*. Here, the detailed structure of the RC may be crucial, as the *B*_A_ and *B*_B_ BChls are somewhat more deeply buried within the protein structure than the BChls that make up the *P* dimer (see Fig. 6a, which shows the separation between the proximal edges of the *B*_A_/*B*_B_ BChls (dashed line), *P* BChls (dotted line) and the protein surface (solid line)). When viewed from the periplasmic side of the membrane (Fig. 6b), it is apparent that the P BChls are separated from the aqueous phase on the periplasmic side of the membrane by the thickness of a single layer of amino acids, specifically a tyrosine and leucine (highlighted with cyan carbons). In contrast, the *B*_A_ and *B*_B_ BChls are each insulated from the aqueous phase by the diameter of an amphipathic α-helix. There is a physiological significance of this structural arrangement: one function of the RC protein being to insulate the cofactors that form the electron transfer wires from the external phase and so preventing unwanted redox reactions that could compromise the quantum yield of charge separation. Only the *P* dimer and *Q*_B_ ‘termini’ of this wire are exposed to the external redox circuit in the natural electron transfer system, and our findings on electron conduction through the RC shows the same is the case when this complex protein is incorporated into an artificial nanoscale electric circuit, with electron flow between these termini, and hence between the conducting substrate and tip, subject to the same striking asymmetric conductance that governs natural charge separation.

## Methods

### Biological material

Antenna-deficient strains of *Rba. sphaeroides* containing wild-type RCs and the AM260W and AM149W variants were constructed as described previously^40^,^41^,^63^. Cultures of all three strains were grown under semi-aerobic conditions in the dark^64^. Cells harvested by centrifugation were re-suspended in 20 mM Tris (pH 8), lysed using a French pressure cell (Aminco) and RC-rich membranes isolated by ultracentrifugation through a 15%/40% sucrose step gradient, as described in detail previously^64^. Figures describing the structural basis of cofactor exclusion in the AM260W and AM149W RCs were prepared using Protein Data Bank entries 1QOV^42^,^63^ and 2JIY^43^, respectively, with rendering using PyMOL (The PyMOL Molecular Graphics System, Version 1.5.0.4 Schrödinger, LLC.).

### Photochronoamperometry

Semi-transparent working electrodes were prepared by sputtering a thin layer of gold on 25-mm diameter glass coverslips that had been cleaned by sonication in methanol for 1 h, rinsed with milli-Q water, dried in a stream of nitrogen gas and placed in an ozone cleaner for 1 h (PR-100 UV-Ozone Photoreactor, UVP). A 12-nm-thick gold film of was deposited on top of a 1- to 2-nm-thick molybdenum–germanium (MoGe) adhesion layer using a magnetron sputtering system (ATC 1800-F, AJA International)^65^. The sputtering rate was 1.32 nm min^−1^ in a 10-mTorr argon environment for MoGe and 9.06 nm min^−1^ in a mixure of 10-mTorr argon and 1-mTorr oxygen for gold. The gold layers obtained were flat (~2–3 Å root-mean-square roughness), homogenous and conductive over the full area of the coated surface. The gold-coated glass coverslips were stored in a desiccator and used within 1 week of preparation.

RCs were adhered to working electrodes by incubating gold-coated glass coverslips in a 1 mg ml^−1^ solution of RC-rich membranes in 20 mM Tris (pH 8) for 1 h at 4 °C, followed by rinsing with 20 mM Tris (pH 8) to remove any loosely bound membrane fragments. Absorbance spectra of adhered RC membranes were recorded using a fibre-coupled spectrometer (Ocean Optics, HUV4000) equipped with a charge-coupled device array detector and with halogen and deuterium lamps as light sources. Coverage was estimated using an extinction coefficient of 2.88 × 10^5^ M^−1^ cm^−1^ at 802 nm^66^.

Photocurrents were measured using a conventional three-electrode setup, with the coated gold electrode forming the base of the measuring cell. The cell was filled with 20 mM Tris (pH 8) into which was inserted a saturated calomel reference electrode and platinum wire counter electrode. Illumination was provided from below by a shuttered LED with a central wavelength of 880 nm and a bandwidth of 50 nm. The LED was operated at 800 mA and 7.6 V, and the intensity of light reaching the surface of the working electrode was 23 mW cm^−2^. Action spectra were recorded using white light passed through a monochromator; the light intensity at the surface of the working electrode was 2 mW cm^−2^ at 880 nm. The wavelength of the excitation light was changed in steps of 5 nm and illumination was turned on for 10 s at every probe wavelength. Ubiquinone-0 and horse heart cytochrome *c* were used as redox mediators.

### Conductive AFM

For C-AFM, a solution of RC-rich membrane fragments (1 mg RCs per ml) was spread on the surface of the water in an LB trough (KSV Minitrough, KSV Instruments Ltd, Helsinki, Finland) and after equilibration a surface-assembled membrane layer was compressed to a surface pressure of 35 mN m^−1^. A 25-mm diameter gold-coated glass coverslip was vertically dipped into the water sub-phase at a speed of 1 mm min^−1^ and a constant surface pressure. The topography of RC-rich membrane fragments deposited in this manner was investigated using a Nanoscope IIIa AFM (Veeco, USA) in tapping mode under ambient conditions. Standard silicon nitride cantilevers with resonant frequency of 75 kHz and force constant of 2 N m^−1^ were used for imaging.

Current–voltage characteristics were measured at a junction fabricated by sandwiching the layer of adhered RC-rich membranes between a C-AFM tip and the gold surface. To make them conductive, standard silicon nitride AFM probes were coated with a thin layer of platinum, using a magnetron sputtering system (ATC 1800-F, AJA International). The radius of curvature of the probe after sputtering was ~15 nm as assessed by scanning electron microscopy. Tapping mode AFM imaging was used to locate the RC complexes, after which the tip was positioned on top of a patch of RC-rich membranes with control of the applied force. A voltage ramp was applied between the tip and substrate, and the resulting current was measured. A current-to-voltage converter, mounted very close to the C-AFM tip, limited current measurements to values of less than ±10 nA.

## Additional information

**How to cite this article:** Kamran, M. *et al*. Demonstration of asymmetric electron conduction in pseudosymmetrical photosynthetic reaction centre proteins in an electrical circuit. *Nat. Commun*. 6:6530 doi: 10.1038/ncomms7530 (2015).

## References

[b1] Davis J. J. . Molecular bioelectronics. J. Mat. Chem. 15, 2160–2174 (2005) .

[b2] Ron I., Pecht I., Sheves M. & Cahen D. Proteins as solid-state electronic conductors. Acc. Chem. Res. 43, 945–953 (2010) . 20329769 10.1021/ar900161u

[b3] Sek S. Peptides and proteins wired into the electrical circuits: An SPM-based approach. Biopolymers 100, 71–81 (2013) . 23335169 10.1002/bip.22148

[b4] Zhang J. D. . Single-molecule electron transfer in electrochemical environments. Chem. Rev. 108, 2737–2791 (2008) . 18620372 10.1021/cr068073+

[b5] Nitzan A. & Ratner M. A. Electron transport in molecular wire junctions. Science 300, 1384–1389 (2003) . 12775831 10.1126/science.1081572

[b6] Patil A. V. & Davis J. J. Molecular scale bioelectrochemistry. Coord. Chem. Rev. 255, 1970–1980 (2011) .

[b7] Gerster D. . Photocurrent of a single photosynthetic protein. Nat. Nanotechnol. 7, 673–676 (2012) . 23023644 10.1038/nnano.2012.165

[b8] Feher G., Allen J. P., Okamura M. Y. & Rees D. C. Structure and function of bacterial photosynthetic reaction centers. Nature 339, 111–116 (1989) .

[b9] Hoff A. J. & Deisenhofer J. Photophysics of photosynthesis. Structure and spectroscopy of reaction centers of purple bacteria. Phys. Rep. 287, 1–247 (1997) .

[b10] Jones M. R. The petite purple photosynthetic powerpack. Biochem. Soc. Trans. 37, 400–407 (2009) . 19290870 10.1042/BST0370400

[b11] van Rotterdam B. J., Crielaard W., van Stokkum I. H. M., Hellingwerf K. J. & Westerhoff H. V. Simplicity in complexity: the photosynthetic reaction center performs as a simple 0.2 V battery. FEBS Lett. 510, 105–107 (2002) . 11755540 10.1016/s0014-5793(01)03210-0

[b12] Yehezkeli O., Tel-Vered R., Michaeli D., Willner I. & Nechushtai R. Photosynthetic reaction center-functionalized electrodes for photo-bioelectrochemical cells. Photosynth. Res. 120, 71–85 (2014) . 23371753 10.1007/s11120-013-9796-3

[b13] Zaitsev S. Y., Solovyeva D. O. & Nabiev I. R. Nanobiohybrid structures based on the organized films of photosensitive membrane proteins. Russ. Chem. Rev. 83, 38–81 (2014) .

[b14] Boghossian A. A., Ham M.-H., Choi J. H. & Strano M. S. Biomimetic strategies for solar energy conversion: a technical perspective. Energy Environ. Sci. 4, 3834–3843 (2011) .

[b15] den Hollander M. J. . Enhanced photocurrent generation by photosynthetic bacterial reaction centers through molecular relays, light-harvesting complexes, and direct protein-gold interactions. Langmuir 27, 10282–10294 (2011) . 21728318 10.1021/la2013528

[b16] Tan S. C., Crouch L. I., Jones M. R. & Welland M. Generation of alternating current in response to discontinuous illumination by photoelectrochemical cells based on photosynthetic proteins. Angew. Chem. Int. Ed. 51, 6667–6671 (2012) . 10.1002/anie.201200466 22623225

[b17] Swainsbury D. J., Friebe V. M., Frese R. N. & Jones M. R. Evaluation of a biohybrid photoelectrochemical cell employing the purple bacterial reaction centre as a biosensor for herbicides. Biosens. Bioelectron. 58, 172–178 (2014) . 24637165 10.1016/j.bios.2014.02.050 PMC4009402

[b18] Stamouli A., Frenken J. W., Oosterkamp T. H., Cogdell R. J. & Aartsma T. J. The electron conduction of photosynthetic protein complexes embedded in a membrane. FEBS Lett. 560, 109–114 (2004) . 14988007 10.1016/S0014-5793(04)00080-8

[b19] Mikayama T., Miyashita T., Iida K., Suemori Y. & Nango M. Electron transfer mediated by photosynthetic reaction center proteins between two chemical-modified metal electrodes. Mol. Cryst. Liquid Cryst. 445, 291–296 (2006) .

[b20] Reiss B. D., Hanson D. K. & Firestone M. A. Evaluation of the photosynthetic reaction center protein for potential use as a bioelectronic circuit element. Biotechnol. Prog. 23, 985–989 (2007) . 17625910 10.1021/bp070042s

[b21] Kondo M. . Photocurrent and electronic activities of oriented-His-tagged photosynthetic light-harvesting/reaction center core complexes assembled onto a gold electrode. Biomacromolecules 13, 432–438 (2012) . 22239547 10.1021/bm201457s

[b22] Sumino A., Dewa T., Sasaki N., Kondo M. & Nango M. Electron conduction and photocurrent generation of a light-harvesting/reaction center core complex in lipid membrane environments. J. Phys. Chem. Lett. 4, 1087–1092 (2013) . 26282025 10.1021/jz301976z

[b23] Lee I., Lee J. W., Warmack R. J., Allison D. P. & Greenbaum E. Molecular electronics of a single photosystem-I reaction-center—studies with scanning-tunneling-microscopy and spectroscopy. Proc. Natl Acad. Sci. USA 92, 1965–1969 (1995) . 11607515 10.1073/pnas.92.6.1965 PMC42403

[b24] Lee I., Lee J. W. & Greenbaum E. Biomolecular electronics: vectorial arrays of photosynthetic reaction centers. Phys. Rev. Lett. 79, 3294–3297 (1997) .

[b25] Deisenhofer J., Epp O., Miki K., Huber R. & Michel H. Structure of the protein subunits in the photosynthetic reaction center of *Rhodopseudomonas viridis* at 3 A˚ resolution. Nature 318, 618–624 (1985) . 22439175 10.1038/318618a0

[b26] Ferreira K. N., Iverson T. M., Maghlaoui K., Barber J. & Iwata S. Architecture of the photosynthetic oxygen-evolving center. Science 303, 1831–1838 (2004) . 14764885 10.1126/science.1093087

[b27] Jordan P. . Three-dimensional structure of cyanobacterial photosystem I at 2.5 angstrom resolution. Nature 411, 909–917 (2001) . 11418848 10.1038/35082000

[b28] Zouni A. . Crystal structure of photosystem II from *Synechococcus elongatus* at 3.8 angstrom resolution. Nature 409, 739–743 (2001) . 11217865 10.1038/35055589

[b29] Allen J. P., Feher G., Yeates T. O., Komiya H. & Rees D. C. Structure of the reaction center from *Rhodobacter sphaeroides* R-26—the cofactors. Proc. Natl Acad. Sci. USA 84, 5730–5734 (1987) . 3303032 10.1073/pnas.84.16.5730 PMC298936

[b30] Allen J. P., Feher G., Yeates T. O., Komiya H. & Rees D. C. Structure of the reaction center from *Rhodobacter sphaeroides* R-26—the protein subunits. Proc. Natl Acad. Sci. USA 84, 6162–6166 (1987) . 2819866 10.1073/pnas.84.17.6162 PMC299029

[b31] Komiya H., Yeates T. O., Rees D. C., Allen J. P. & Feher G. Structure of the reaction center from *Rhodobacter sphaeroides* R-26 and 2.4.1—symmetry-relations and sequence comparisons between different species. Proc. Natl Acad. Sci. USA 85, 9012–9016 (1988) . 3057498 10.1073/pnas.85.23.9012 PMC282652

[b32] Michel-Beyerle M. E. . Unidirectionality of charge separation in reaction centers of photosynthetic bacteria. Biochim. Biophys. Acta 932, 52–70 (1988) .

[b33] Okamura M. Y., Paddock M. L., Graige M. S. & Feher G. Proton and electron transfer in bacterial reaction centers. Biochim. Biophys. Acta 1458, 148–163 (2000) . 10812030 10.1016/s0005-2728(00)00065-7

[b34] Wraight C. A. Proton and electron transfer in the acceptor quinone complex of photosynthetic reaction centers from *Rhodobacter sphaeroides*. Front. Biosci. 9, 309–337 (2004) . 14766369 10.2741/1236

[b35] Remy A. & Gerwert K. Coupling of light-induced electron transfer to proton uptake in photosynthesis. Nat. Struct. Biol. 10, 637–644 (2003) . 12872158 10.1038/nsb954

[b36] Heller B. A., Holten D. & Kirmaier C. Control of electron-transfer between the L-side and M-side of photosynthetic reaction centers. Science 269, 940–945 (1995) . 7638616 10.1126/science.7638616

[b37] Wakeham M. C. & Jones M. R. Rewiring photosynthesis: engineering wrong-way electron transfer in the purple bacterial reaction centre. Biochem. Soc. Trans. 33, 851–857 (2005) . 16042613 10.1042/BST0330851

[b38] Harris M. A. . Protein influence on charge-asymmetry of the primary donor in photosynthetic bacterial reaction centers containing a heterodimer: effects on photophysical properties and electron transfer. J. Phys. Chem. B 117, 4028–4041 (2013) . 23560569 10.1021/jp401138h

[b39] Williams J. C., Steiner L. A., Feher G. & Simon M. I. Primary structure of the L-subunit of the reaction center from *Rhodopseudomonas sphaeroides*. Proc. Natl Acad. Sci. USA 81, 7303–7307 (1984) . 6095283 10.1073/pnas.81.23.7303 PMC392134

[b40] Ridge J. P., van Brederode M. E., Goodwin M. G., van Grondelle R. & Jones M. R. Mutations that modify or exclude binding of the Q_A_ ubiquinone and carotenoid in the reaction center from *Rhodobacter sphaeroides*. Photosynth. Res. 59, 9–26 (1999) .

[b41] Watson A. J. . Replacement or exclusion of the B-branch bacteriopheophytin in the purple bacterial reaction centre: the H_B_ cofactor is not required for assembly or core function of the *Rhodobacter sphaeroides* complex. Biochim. Biophys. Acta Bioenerg. 1710, 34–46 (2005) . 10.1016/j.bbabio.2005.08.005 16181607

[b42] McAuley K. E. . Ubiquinone binding, ubiquinone exclusion, and detailed cofactor conformation in a mutant bacterial reaction center. Biochemistry 39, 15032–15043 (2000) . 11106481 10.1021/bi000557r

[b43] Fyfe P. K. . Structural responses to cavity-creating mutations in an integral membrane protein. Biochemistry 46, 10461–10472 (2007) . 17711306 10.1021/bi701085w

[b44] Magis G. J. . Light harvesting, energy transfer and electron cycling of a native photosynthetic membrane adsorbed onto a gold surface. Biochim. Biophys. Acta 1798, 637–645 (2010) . 20036635 10.1016/j.bbamem.2009.12.018

[b45] Kamran M., Delgado J. D., Friebe V., Aartsma T. J. & Frese R. N. Photosynthetic protein complexes as bio-photovoltaic building blocks retaining a high internal quantum efficiency. Biomacromolecules 15, 2833–2838 (2014) . 24964245 10.1021/bm500585s

[b46] Zhao J. W., Davis J. J., Sansom M. S. P. & Hung A. Exploring the electronic and mechanical properties of protein using conducting atomic force microscopy. J. Am. Chem. Soc. 126, 5601–5609 (2004) . 15113232 10.1021/ja039392a

[b47] Das R. . Integration of photosynthetic protein molecular complexes in solid-state electronic devices. Nano Letts 4, 1079–1083 (2004) .

[b48] Lu Y., Xu J., Liu B. & Kong J. Photosynthetic reaction center functionalized nano-composite films: Effective strategies for probing and exploiting the photo-induced electron transfer of photosensitive membrane protein. Biosens. Bioelectron. 22, 1173–1185 (2007) . 16815004 10.1016/j.bios.2006.05.026

[b49] Lukashev E. P., Nadtochenko V. A., Permenova E. P., Sarkisov O. M. & Rubin A. B. Electron phototransfer between photosynthetic reaction centers of the bacteria *Rhodobacter sphaeroides* and semiconductor mesoporous TiO_2_ films. Dokl. Biochem. Biophys. 415, 211–216 (2007) . 17933338 10.1134/s1607672907040138

[b50] Trammell S. A. . Effects of distance and driving force on photoinduced electron transfer between photosynthetic reaction centers and gold electrodes. J. Phys. Chem. C 111, 17122–17130 (2007) .

[b51] Tan S. C., Crouch L. I., Mahajan S., Jones M. R. & Welland M. E. Increasing the open circuit voltage of photoprotein-based photoelectrochemical cells by manipulation of the vacuum potential of the electrolytes. ACS Nano 6, 9103–9109 (2012) . 23009071 10.1021/nn303333e

[b52] Harrold J. W. Jr. . Functional interfacing of *Rhodospirillum rubrum* chromatophores to a conducting support for capture and conversion of solar energy. J. Phys. Chem. B 117, 11249–11259 (2013) . 23789750 10.1021/jp402108s

[b53] Mirvakili S. M. . Photoactive electrodes incorporating electrosprayed bacterial reaction centers. Adv. Funct. Mater. 24, 4789–4794 (2014) .

[b54] Ishikita H. . Tuning electron transfer by ester-group of chlorophylls in bacterial photosynthetic reaction center. FEBS Lett. 579, 712–716 (2005) . 15670833 10.1016/j.febslet.2004.12.049

[b55] Steffen M. A., Lao K. Q. & Boxer S. G. Dielectric asymmetry in the photosynthetic reaction center. Science 264, 810–816 (1994) . 17794722 10.1126/science.264.5160.810

[b56] Gunner M. R., Nicholls A. & Honig B. Electrostatic potentials in *Rhodopseudomonas viridis* reaction centers: implications for the driving force and directionality of electron transfer. J. Phys. Chem. 100, 4277–4291 (1996) .

[b57] Alegria G. & Dutton P. L. Langmuir-Blodgett monolayer films of bacterial photosynthetic membranes and isolated reaction centers—preparation, spectrophotometric and electrochemical characterization. Biochim. Biophys. Acta 1057, 239–257 (1991) . 1849739 10.1016/s0005-2728(05)80107-0

[b58] Miyake J. & Hara M. Protein-based nanotechnology: molecular construction of proteins. Mat. Sci. Engineer. C 4, 213–219 (1997) .

[b59] Nicolini C., Erokhin V., Antolini F., Catasti P. & Facci P. Thermal-stability of protein secondary structure in Langmuir-Blodgett films. Biochim. Biophys. Acta 1158, 273–278 (1993) . 8251527 10.1016/0304-4165(93)90025-4

[b60] Jones M. R. . Mutants of *Rhodobacter sphaeroides* lacking one or more pigment protein complexes and complementation with reaction-center, LH1, and LH2 genes. Mol. Microbiol. 6, 1173–1184 (1992) . 1588816 10.1111/j.1365-2958.1992.tb01556.x

[b61] Mar T., Picorel R. & Gingras G. Phototrapping of doubly reduced monomeric bacteriochlorophyll in the photoreaction center of *Ectothiorhodospira sp*. Biochemistry 32, 1466–1470 (1993) . 8381662 10.1021/bi00057a009

[b62] Mar T. & Gingras G. Origin of optical-activity in the purple bacterial photoreaction center. Biochemistry 34, 9071–9078 (1995) . 7619806 10.1021/bi00028a016

[b63] McAuley K. E. . Structural details of an interaction between cardiolipin and an integral membrane protein. Proc. Natl Acad. Sci. USA 96, 14706–14711 (1999) . 10611277 10.1073/pnas.96.26.14706 PMC24712

[b64] Jones M. R., Heer-Dawson M., Mattioli T. A., Hunter C. N. & Robert B. Site-Specific mutagenesis of the reaction center from *Rhodobacter sphaeroides* studied by Fourier transform Raman spectroscopy—mutations at tyrosine M210 do not affect the electronic-structure of the primary donor. FEBS Lett. 339, 18–24 (1994) . 8313970 10.1016/0014-5793(94)80376-5

[b65] van Baarle G. J. C., Troianovski A. M., Nishizaki T., Kes P. H. & Aarts J. Imaging of vortex configurations in thin films by scanning-tunneling microscopy. Appl. Phys. Lett. 82, 1081–1083 (2003) .

[b66] Straley S. C., Parson W. W., Mauzerall D. C. & Clayton R. K. Pigment content and molar extinction coefficients of photochemical reaction centers from *Rhodopseudomonas spheroides*. Biochim. Biophys. Acta 305, 597–609 (1973) . 4354794 10.1016/0005-2728(73)90079-0

